# Supplementary material for: Integrating Molecular Alterations with Immunophenotype and Clinical Characteristics in Myelodysplastic Syndromes: A Single-Center Study
Source: Int J Mol Sci. 2025 Jul 30;26(15):7382. doi: 10.3390/ijms26157382 (PMC12347109; doi:10.3390/ijms26157382)
Supplement: Supplementary file 1 [file ijms-26-07382-s001.zip › ijms-3714525-supplementary.pdf]

Table S1. Patients mutational status.

Patient ID	Gene	Nucleotide ID	VAF %	Protein ID	Variant classification	Frequency (gnomAD, dbSNP	COSMIC	ELN 2012 score	ELN 2012+ 11b/DR +13/DR
1	*KIT*	c.2447A>T	15	p.Asp816Val	Pathogenic	0.00005	COSV55386424	2	2
	KRAS	c.436G>A	10	p.Ala146Thr	Pathogenic	0	COSV55501778		
	TET2	c.2662C>T	47,4	p.Gln888Ter	Likely pathogenic	0.00000399	COSV54417495		
	TET2	c.4124T>G	45,4	p.Leu1375Trp	VUS	0	N/A		
	U2AF1	c.101C>T	45,7	p.Ser34Phe	Pathogenic	0.0000319	COSV52341059		
2	*DNMT3A*	c.2185C>T	35,8	p.Arg729Trp	VUS	0.00004079	COSV53036577	3	4
	NPM1	c.863_864insCATG	33,7	Trp288CysfsTer12	Pathogenic	0	COSV51542987		
3	*TET2*	c.4133G>A	44,2	p.Cys1378Tyr	VUS	0	COSV54395999	3	4
	TET2	c.4707C>G	45,5	p.Tyr1569Ter	Likely pathogenic	0	N/A		
	NPM1	c.860_863dup	40,4	p.Trp288CysfsTer12	Pathogenic	0.000008	COSV51542664		
	RUNX1	c.234G>A	51,1	p.Met78Ile	VUS	0	N/A		
4	*DNMT3A*	c.1342del	40,1	p.Tyr448ThrfsTer203	likely pathogenic	0	N/A	4	5
	GATA2	c.1114G>A	10,1	p.Ala372Thr	Likely pathogenic/VUS	0	COSV62002954		
	SRSF2	c.284C>G	42,3	p.Pro95Arg	Likely pathogenic	0.0000292	COSV57969809		
	STAG2	c.1165C>T	83,9	p.Gln389Ter	Likely pathogenic	0	COSV54356658		
5	*JAK2*	c.1849G>T	37,2	p.Val617Phe	Pathogenic	0.0003444	COSV67569051	2	4
	TP53	c.377A>G	35,8	p.Tyr126Cys	Pathogenic/VUS	0	COSV52689293		
	TP53	c.858del	35,2	p.Glu287ArgfsTer58	Likely pathogenic	0	N/A		
6	*TP53*	c.488A>G	91	p.Tyr163Cys	Pathogenic	0	COSV52663142	2	4
	NRAS	c.35G>A	12,5	p.Gly12Asp	Pathogenic/Likely pathogenic	0.000007953	COSV54736383		
7	*RUNX1*	c.292del	48,7	p.Leu98SerfsTer24	Pathogenic	0	COSV55867617	3	3
	ASXL1	c.1900_1922del	47,4	p.Glu635ArgfsTer15	Pathogenic	0.00006145	COSV60102280		
	U2AF1	c.472_477dup	45,5	p.Tyr158_Glu159dup	VUS	0.000003978	COSV52342397		
8	*KIT*	c.2447A>T	39	p.Asp816Val	Pathogenic	0.00005	COSV55386424	2	3
	TP53	c.707A>G	31,7	p.Tyr236Cys	Pathogenic	0	COSV52662150		

	TP53	c.713G>A	52,3	p.Cys238Tyr	Pathogenic/Likely pathogenic	0.000007953	COSV52661646		
9	*RUNX1*	c.317G>C	20,1	p.Trp106Ser	Likely pathogenic/Pathogenic	0	COSV55871891	3	3
	RUNX1	c.334C>G	70,2	p.Leu112Val	Likely pathogenic/Pathogenic	0	N/A		
	ASXL1	c.2074C>T	45,5	p.Gln692Ter	Likely pathogennic	0	COSV60104922		
	PRPF8	c.1253C>G	49	p.Thr418Ser	VUS	0.0002027	N/A		
	PHF6	c.821G>A	82,4	p.Arg274Gln	VUS/Patogenny	0	COSV59699120		
10	*TET2*	c.3965T>A	46,4	p.Leu1322Gln	VUS	0	COSV54406495	3	3
	TET2	c.840dup	46,6	p.Asn281Ter	Likely pathogennic	0.000007999	COSV54400450		
	NRAS	c.179G>A	49,8	p.Gly60Glu	Pathogenic	0.00003186	COSV54736394		
	ASXL1	c.1934dup	35	p.Gly646TrpfsTer12	Pathogenic/Likely pathogenic	0.0004276	COSV60102155		
	SRSF2	c.284C>T	28,7	p.Pro95Leu	Pathogenic	0.00006264	COSV57969830		
11	*IDH1*	c.394C>T	51	p.Arg132Cys	Pathogenic	0	COSV61615256	3	4
	RUNX1	c.1078_1082dup	48,7	p.Gly363ProfsTer233	Likely pathogennic	0	N/A		
	ASXL1	c.1936_1937insC	47,6	p.Gly646AlafsTer12	Likely pathogennic	0	N/A		
12	*DNMT3A*	c.1628G>C	25	p.(Gly543Ala)	Likely pathogenic	0.000003977	COSV53051884	3	4
13	*TET2*	c.3819T>A	43,1	p.Cys1273Ter	Likely pathogenic	0	N/A	3	5
	TET2	c.4099C>T	45,9	p.Pro1367Ser	VUS	0	N/A		
	NF1	c.289-6T>C	49,8	Splice region	Likely benign/VUS	0.000003987	N/A		
	ZRSR2	c.524A>G	85	p.Tyr175Cys	VUS	0	N/A		
	PHF6	c.418+1G>A	81	Splice donor	Likely pathogenic	0	N/A		
14	*ASXL1*	c.1934dup	30,6	p.Gly646TrpfsTer12	Pathogenic/Likely pathogenic	0.0004276	COSV60102155	4	4
15	*TP53*	c.844C>T	21	p.Arg282Trp	Pathogenic	0.000003978	COSV52662048	3	3
	TP53	c.524G>A	21,8	p.Arg175His	Pathogenic	0.000003980	COSV52661038		
16	-	-	-	-	-	-	-	3	4
17	*CSF3R*	c.1853C>T	23,2	p.Thr618Ile	Pathogenic	0	COSV58963463	3	4
	ASXL1	c.1900_1922del	6,9	p.Glu635ArgfsTer15	Pathogenic	0.00006145	COSV60102280		

**18**	*TET2*	c.3594+5G>A	45,9	splice region	VUS	0	N/A	2	2
	TET2	c.5566del	52,2	p.Asp1856IlefsTer31	Likely pathogenic	0	N/A		
**19**	*TP53*	c.814G>A	84,5	p.Val272Met	Pathogenic	0.000003986	COSV52661812	2	4
**20**	*SRSF2*	c.284C>A	42	p.Pro95His	VUS	0.0001034	COSV57969816	3	4
	MPL	c.1775G>A	50	p.Arg592Gln	VUS	0.00001768	COSV65244459		
	ASXL1	1900_1922del	39,6	p.Glu635ArgfsTer15	Pathogenic	0.00006145	COSV60102280		
	STAG2	c.1810C>T	9,7	p.Arg604Ter	Likely Pathogenic	0	COSV54356853		
	STAG2	c.1907dup	10,1	Tyr636Ter	Likely Pathogenic	0	COSV54351126		
	STAG2	c.2025+1G>T	20,5	splice donor	Likely Pathogenic	0	N/A		
**21**	-	-	-	-	-	-	-	1	3
**22**	*TET2*	c.4729_4756del	69,4	p.Ser1577GlnfsTer10	Likely Pathogenic	0	N/A	3	5
	SF3B1	c.2098A>G	37,4	p.Lys700Glu	Likely Pathogenic	0.00008314	COSV59205318		
**23**	*ASXL1*	c.1934dup	34,2	p.Gly646TrpfsTer12	Pathogenic	0.0004276	COSV60102155	2	3
	TET2	c.822del	36,5	p.Asn275IlefsTer18	Pathogenic	0.00001673	N/A		
	STAG2	c.386-8A>G	73,8	Splice region	VUS	0	N/A		
	SRSF2	c.284C>T	31,4	p.Pro95Leu	Likely Pathogenic	0.00005090	COSV57969830		
**24**	*TET2*	c.822del	42,7	p.Asn275IlefsTer18	Pathogenic	0.00001673	N/A	2	4
	TET2	c.3851C>A	46,1	p.Ser1284Tyr	VUS	0	N/A		
	CBL	c.1258C>G	78,2	p.Arg420Gly	Likely pathogenic	0	COSV50630874		
**25**	*TET2*	c.444_447del	42,7	p.Lys148AsnfsTer3	Likely pathogenic	0	COSV105837197	4	6
	RUNX1	c.806-1_806dup	39,7	p.Asp269GlufsTer43/splice region	Likely pathogenic	0	N/A		
	ASXL1	c.1934dup	36,1	p.Gly646TrpfsTer12	Pathogenic	0.0004276	COSV60102155		
	STAG2	c.3034C>T	38,8	p.Arg1012Ter	Pathogenic	0	COSV54350913		
	SRSF2	c.284C>A	42,9	p.Pro95His	VUS	0.0001034	COSV57969816		
**26**	*ASXL1*	c.1934dup	35,2	p.Gly646TrpfsTer12	Pathogenic	0.0004276	COSV60102155	2	4
	IKZF1	c.7G>A	49,5	p.Ala3Thr	VUS	0.000001240	N/A		
	STAG2	c.3034C>T	27,9	p.Arg1012Ter	Pathogenic	0	COSV54350913		
	RUNX1	c.766_767del	26,6	Ser256HisfsTer4	Likely pathogenic	0	N/A		
	SRSF2	c.284C>A	34,2	p.Pro95His	VUS	0.0001034	COSV57969816		
**27**	*RUNX1*	c.1006T>A	45,2	p.Phe336Ile	VUS	0.00001433	N/A	2	4

	STAG2	c.2776-4del	9,2	Splice region	VUS	0	N/A		
**28**								2	4
**29**	*SF3B1*	c.2098A>G	31,8	p.Lys700Glu	Likely Pathogenic	0.00004489	COSV59205318	4	6
	STAG2	c.2265+1G>C	18,2	Splice donor	VUS/Pathogenic	0	N/A		
**30**	*TET2*	c.1968del	38,6	p.Ser657HisfsTer43	Likely pathogenic	0.000001859	N/A	4	4
	TET2	c.4140T>G	45,8	p.His1380Gln	VUS	0.000001289	N/A		

VAF- Variant allele frequency VUS- Variants of uncertain significance.

Table S2. The results of individual parameters within the ELN 2012 score.

Patient number	Number of CD34+ cells	Number of Nucleated cell (P1)	Percentage of B progenitors	Percentage of myeloblasts	CD 45 MFI on Lymphocytes	CD 45 MFI on Myeloblasts	CD45 MFI ratio	Granulocytes SSC	Lyphomcytes SSC	SSC ratio
1	129	48047	2.32558	0.27	11169	2055	5.43504	84480	18688	4.52055
2	524	160060	2.67176	0.32	7158	2268	3.15608	32000	8448	3.78788
3	584	95259	0.68493	0.61	9323	2760	3.3779	26368	10240	2.575
4	1895	34406	2.05805	5.39	8315	802	10.3678	30208	7680	3.93333
5	2224	153997	0.89928	1.43	5489	928	5.91487	22784	7936	2.87097
6	9559	60706	9.18506	14.30	7735	1798	4.302	25856	8960	2.88571
7	340	74190	0	0.46	9730	1091	8.91842	104192	20224	5.1519
8	4550	24950	8.83516	16.63	7973	1723	4.62739	22272	14080	1.58182
9	10542	80746	0.07589	13.05	11201	1616	6.93131	26112	11008	2.37209
10	2565	551732	2.76803	0.45	8181	2810	2.91139	41472	9981	4.15509
11	6813	163240	1.84941	4.10	9043	1395	6.48244	27136	11008	2.46512
12	12417	177647	0.88588	6.93	8598	1118	7.6905	64768	8960	7.2286
13	3594	325949	0.1113	4.70	3823	973	3.92909	30464	9984	2.46512
14	8264	270743	0.20571	3.05	6802	667	10.1979	35328	11008	3.2093
15	3486	110598	1.52037	3.10	5244	1332	3.93694	66048	8960	7.37143
16	5586	76823	2.72109	7.07	7029	2203	3.19065	62720	7424	8.44828
17	8634	114145	1.9458	7.42	9616	2110	4.55735	79872	14592	5.47368
18	553	152544	3.07414	0.35	8868	1552	5.71392	62720	12032	5.21277
19	351	35061	1.7094	0.98	5519	832	6.63341	33536	13056	2.56863
20	5749	302967	2.19169	1.86	5071	1358	3.73417	22784	7168	3.17857
21	2487	308885	8.92642	0.88	7408	1396	5.30659	51968	11008	4.72093
22	7297	144956	0.6578	5.00	8185	1316	6.2196	25344	10496	2.41463
23	138	264988	8.69565	0.05	4310	3805	1.13272	6400	7936	0.80645

24	4479	288360	4.15271	1.49	7657	861	8.89315	63488	10496	6.04878
25	7355	109445	0.17675	6.71	7308	788	9.27411	43776	7936	5.51613
26	616	139685	0.48701	0.44	5766	1285	4.48716	33536	7936	4.22581
27	1355	206922	1.54982	0.64	4440	778	5.70694	26624	9728	2.73684
28	9529	333591	9.66523	2.58	5019	741	6.7733	25344	13312	1.9038
29	21054	545964	0.47497	3.84	2999	851	3.5241	54528	9472	5.7568
30	36234	452799	1.94845	7.85	12441	3443	3.6134	78592	16896	4.6515

MFI- mean fluorescensce index, SSC- side scatter count.

Table S3. Results of Non-Parametric Statistical Tests Evaluating the Relationships Between Genetic Mutations and Clinical or Hematologic Parameters.

Parameter 1	Parameter 2	Mann-Whitney Test p value	Permutation t-test p value	Kendall Tau Correlation p value	Welch's t-test p value
**Cohesin complex mutation**	R-IPSS value	0.44	0.45	n/a	n/a
**Cohesin complex mutation**	Concentration of hemoglobin	1	0.91	n/a	n/a
**Epigenetic regulation mutation**	R-IPSS value	0.45	0.36	n/a	0.38
**Epigenetic regulation mutation**	Concentration of hemoglobin	0.89	0.96	n/a	0.96
**Epigenetic regulation mutation**	Platelets count	0.91	0.95	n/a	n/a
**Epigenetic regulation mutation**	White blood cell count	0.38	0.22	n/a	n/a
**Epigenetic regulation mutation**	ELN 2012 score	**0.03**	**0.04**	n/a	n/a
**Epigenetic regulation mutation**	Extended ELN 2012 score	0.50	0.40	n/a	0.40
**Epigenetic regulation pathogenic mutation**	R-IPSS value	0.45	0.37	n/a	0.38
**Epigenetic regulation pathogenic mutation**	Concentration of hemoglobin	0.89	0.96	n/a	0.96
**Epigenetic regulation pathogenic mutation**	Platelets count	0.91	0.95	n/a	n/a
**Epigenetic regulation pathogenic mutation**	White blood cell count	0.38	0.22	n/a	n/a
**Epigenetic regulation pathogenic mutation**	ELN 2012 score	**0.03**	**0.05**	n/a	n/a
**Epigenetic regulation pathogenic mutation**	Extended ELN 2012 score	0.50	0.40	n/a	0.40
**mRNA splicing mutation**	R-IPSS value	0.32	0.24	n/a	n/a
**mRNA splicing mutation**	Concentration of hemoglobin	0.62	0.84	n/a	0.83
**mRNA splicing mutation**	Platelets count	0.51	0.47	n/a	0.47
**mRNA splicing mutation**	White blood cell count	1	0.68	n/a	n/a
**mRNA splicing mutation**	Extended ELN 2012 score	0.53	0.56	n/a	0.56
**mRNA splicing mutation**	ELN 2012 score	0.60	0.49	n/a	n/a
**Cohesin complex mutation**	White blood cell count	0.07	**0.03**	n/a	n/a
**Cohesin complex mutation**	Platelets count	0.46	0.67	n/a	n/a
**Cohesin complex mutation**	Extended ELN 2012 score	0.06	0.05	n/a	n/a
**Cohesin complex mutation**	ELN 2012 score	0.79	0.93	n/a	n/a
**Cohesin complex pathogenic mutation**	R-IPSS value	0.41	0.33	n/a	n/a
**Cohesin complex pathogenic mutation**	Concentration of hemoglobin	0.90	0.79	n/a	n/a
**Cohesin complex pathogenic mutation**	Platelets count	0.55	0.37	n/a	n/a
**Cohesin complex pathogenic mutation**	White blood cell count	0.39	0.16	n/a	n/a
**Cohesin complex pathogenic mutation**	Extended ELN 2012 score	0.26	0.11	n/a	n/a
**Cohesin complex pathogenic mutation**	ELN 2012 score	0.97	0.85	n/a	n/a
**mRNA splicing pathogenic mutation**	R-IPSS value	0.35	0.38	n/a	n/a

**mRNA splicing pathogenic mutation**	Concentration of hemoglobin	0.61	1.00	n/a	n/a
**mRNA splicing pathogenic mutation**	Platelets count	0.19	0.21	n/a	n/a
**mRNA splicing pathogenic mutation**	White blood cell count	0.61	0.89	n/a	n/a
**mRNA splicing pathogenic mutation**	Extended ELN 2012 score	0.39	0.58	n/a	n/a
**mRNA splicing pathogenic mutation**	ELN 2012 score	0.42	0.40	n/a	n/a
**Number of pathogenic mutations**	Extended ELN 2012 score	n/a	n/a	0.84	n/a
**Number of pathogenic mutations**	Concentration of hemoglobin	n/a	n/a	0.09	n/a
**Number of pathogenic mutations**	Platelets count	n/a	n/a	0.13	n/a
**Number of pathogenic mutations**	ELN 2012 score	n/a	n/a	0.66	n/a
**Number of pathogenic mutations**	R-IPSS value	n/a	n/a	0.82	n/a
**Number of pathogenic mutations**	White blood cell count	n/a	n/a	0.26	n/a
**Signal pathway mutation**	R-IPSS value	0.76	0.84	n/a	n/a
**Signal pathway mutation**	Platelets count	0.05	**0.03**	n/a	n/a
**Signal pathway mutation**	Concentration of hemoglobin	0.26	0.39	n/a	n/a
**Signal pathway mutation**	White blood cell count	0.70	0.81	n/a	n/a
**Signal pathway mutation**	Extended ELN 2012 score	0.25	0.14	n/a	n/a
**Signal pathway mutation**	ELN 2012 score	0.60	0.64	n/a	n/a
**Signal pathway pathogenic mutation**	R-IPSS value	0.25	0.30	n/a	n/a
**Signal pathway pathogenic mutation**	Concentration of hemoglobin	0.44	0.49	n/a	n/a
**Signal pathway pathogenic mutation**	Platelets count	0.09	0.06	n/a	n/a
**Signal pathway pathogenic mutation**	White blood cell count	0.31	0.68	n/a	n/a
**Signal pathway pathogenic mutation**	Extended ELN 2012 score	0.19	0.11	n/a	n/a
**Signal pathway pathogenic mutation**	ELN 2012 score	0.46	0.47	n/a	n/a
**Total number of mutations**	Extended ELN 2012 score	n/a	n/a	0.90	n/a
**Total number of mutations**	Concentration of hemoglobin	n/a	n/a	0.21	n/a
**Total number of mutations**	Platelets count	n/a	n/a	0.39	n/a
**Total number of mutations**	ELN 2012 score	n/a	n/a	0.98	n/a
**Total number of mutations**	R-IPSS value	n/a	n/a	0.66	n/a
**Total number of mutations**	White blood cell count	n/a	n/a	0.32	n/a
**Transcriptional factor mutation**	R-IPSS value	0.92	0.93	n/a	n/a
**Transcriptional factor mutation**	Concentration of hemoglobin	0.26	1.04	n/a	n/a
**Transcriptional factor mutation**	Platelets count	0.71	0.53	n/a	n/a
**Transcriptional factor mutation**	White blood cell count	0.10	0.38	n/a	n/a
**Transcriptional factor mutation**	Extended ELN 2012 score	0.84	0.84	n/a	n/a
**Transcriptional factor mutation**	ELN 2012 score	0.85	0.82	n/a	n/a
**Transcriptional factor pathogenic mutation**	R-IPSS value	0.78	0.77	n/a	n/a

**Transcriptional factor pathogenic mutation**	Concentration of hemoglobin	0.08	0.17	n/a	n/a
**Transcriptional factor pathogenic mutation**	Platelets count	0.52	0.50	n/a	n/a
**Transcriptional factor pathogenic mutation**	White blood cell count	0.06	0.34	n/a	n/a
**Transcriptional factor pathogenic mutation**	Extended ELN 2012 score	0.92	0.85	n/a	n/a
**Transcriptional factor pathogenic mutation**	ELN 2012 score	0.53	0.44	n/a	n/a
**Tumor suppressors mutation**	R-IPSS value	0.25	0.18	n/a	n/a
**Tumor suppressors mutation**	Platelets count	0.09	**0.03**	n/a	n/a
**Tumor suppressors mutation**	Concentration of hemoglobin	0.08	0.06	n/a	n/a
**Tumor suppressors mutation**	White blood cell count	0.77	0.23	n/a	n/a
**Tumor suppressors mutation**	Extended ELN 2012 score	0.95	0.86	n/a	n/a
**Tumor suppressors mutation**	ELN 2012 score	0.14	0.08	n/a	n/a
**Tumor suppressor pathogenic mutation**	R-IPSS value	0.25	0.18	n/a	n/a
**Tumor suppressor pathogenic mutation**	Concentration of hemoglobin	0.24	0.20	n/a	n/a
**Tumor suppressor pathogenic mutation**	Platelets count	0.21	0.09	n/a	n/a
**Tumor suppressor pathogenic mutation**	White blood cell count	0.44	0.16	n/a	n/a
**Tumor suppressor pathogenic mutation**	ELN 2012 score	0.07	**0.05**	n/a	n/a
**Tumor suppressor pathogenic mutation**	Extended ELN 2012 score	0.65	0.64	n/a	n/a
**IPSS-M**	ELN 2012 score	n/a	n/a	0.89	n/a
**IPSS-M. descriptive**	ELN 2012 score	n/a	n/a	0.90	n/a
**R-IPSS**	ELN 2012 score	n/a	n/a	0.75	n/a
**R-IPSS value**	ELN 2012 score	n/a	n/a	0.87	n/a

R-IPSS- revised international prognostic score. IPSS-M- molecular international prognostic score.
